# Post-SARS-CoV-2 infection and post-vaccine-related neurological complications share clinical features and the same positivity to anti-ACE2 antibodies

**DOI:** 10.3389/fimmu.2024.1398028

**Published:** 2024-08-01

**Authors:** Margherita Bellucci, Federica Maria Bozzano, Chiara Castellano, Giampaola Pesce, Alessandro Beronio, Alireza Hajabbas Farshchi, Alessandro Limongelli, Antonio Uccelli, Luana Benedetti, Andrea De Maria

**Affiliations:** ^1^ Department of Neuroscience, Rehabilitation, Ophthalmology, Genetics, Maternal and Child Health, University of Genova, Genoa, Italy; ^2^ Istituti di Ricovero e Cura a Carattere Scientifico (IRCCS), Ospedale Policlinico San Martino, Genova, Italy; ^3^ Department of Neurology, Sant’Andrea Hospital, La Spezia, Italy; ^4^ Department of Health Sciences (DISSAL), University of Genova, Genoa, Italy

**Keywords:** anti-idiotype antibodies, anti-ACE2 antibodies, PACS, PCVs, COVID-19

## Abstract

**Introduction:**

A potential overlap in symptoms between post-acute COVID-19 syndrome and post-COVID-19 vaccination syndrome has been noted. We report a paired description of patients presenting with similar manifestations involving the central (CNS) or peripheral nervous system (PNS) following SARS-CoV-2 infection or vaccination, suggesting that both may have triggered similar immune-mediated neurological disorders in the presence of anti-idiotype antibodies directed against the ACE2 protein.

**Patients and methods:**

Four patients exhibited overlapping neurological manifestations following SARS-CoV-2 infection or vaccination: radiculitis, Guillain–Barré syndrome, and MRI-negative myelitis, respectively, sharing positivity for anti-ACE2 antibodies. Autoantibodies against AQP-4, MOG, GlyR, GAD, and amphiphysin, onconeural antibodies for CNS syndromes, and anti-ganglioside antibodies for PNS syndromes tested negative in all patients.

**Discussion:**

Anti-idiotype antibodies against ACE2 have been detected in patients who recovered from COVID-19 infection, and it has been hypothesized that such antibodies may mediate adverse events following SARS-CoV-2 infection or vaccination, resulting in the activation of the immune system against cells expressing ACE2, such as neurons. Our data reveal clinically overlapping syndromes triggered by SARS-CoV-2 infection or vaccination, sharing positivity for anti-ACE2 antibodies. Their presence, in the absence of other classic autoimmune markers of CNS or PNS involvement, suggests that they might play an active role in the context of an aberrant immune response.

**Conclusion:**

Anti-idiotype antibodies directed against ACE2 may be triggered by both SARS-CoV-2 infection and vaccination, possibly contributing to neurological autoimmune manifestations. Their pathogenic role, however, remains to be demonstrated in large-scale, more structured studies.

## 1 Introduction

Following the COVID-19 pandemic outbreak, a broad spectrum of neurological manifestations has been observed in patients infected with severe acute respiratory syndrome coronavirus 2 (SARS-CoV-2) (1, 2). These range from non-specific complications associated with severe disease, such as hypoxic encephalopathy and critical illness myopathy and polyneuropathy, to neurological disorders directly or indirectly caused by the virus, encompassing anosmia, dysgeusia, headache, stroke, Guillain–Barré syndrome (GBS), and infectious, para-infectious, and post-infectious encephalitis (3). These findings raise the possibility that SARS-CoV-2 could underlie a condition of deranged responses to the virus or its components through interaction with or excessive activation of some components of the immune system. Similarly, anti-COVID-19 vaccines can trigger rare but severe immune-mediated side effects, including immune thrombotic thrombocytopenia, myocarditis, and IgA vasculitis (4). Additionally, the neurological side effects of SARS-CoV-2 vaccines have been reported, too, the most prominent being headache, GBS, venous sinus thrombosis, and transverse myelitis (5, 6). Small fiber neuropathy (SFN) has also been described in association with COVID-19 vaccination (7). A number of case reports and small case series have suggested that SFN may also be a possible side effect of COVID-19 vaccination. The symptoms of SFN in these patients included numbness, tingling, and pain in the hands, feet, and face. In some cases, the symptoms were severe enough to interfere with daily activities (8–11).

SARS-CoV-2 infection and COVID-19 vaccination are both associated with some neurological complications and adverse reactions, the most relevant being GBS (6, 12, 13).

According to the Network Hypothesis formulated by Niels Jerne in 1944 (14), the same antibody response to a specific antigen (Ab1) can result in a secondary antibody response (Ab2) directed against the primary antigen-specific antibody. Ab2 antibodies are known as anti-idiotype antibodies and, under physiological conditions, bare a downregulatory role on the immune response. As postulated by Plotz in 1983, anti-idiotype antibodies may underlie the autoimmune response that follows a viral infection (15), as in the case of Coxsackievirus B3 myocarditis (16). Moreover, it has been demonstrated that anti-idiotype antibodies can act as acetylcholine receptor agonists, leading to myasthenia gravis in animal models (17, 18).

Both SARS-CoV-2 virus and COVID-19 vaccine elicit an antibody response against the spike (S) protein, which is used by the coronavirus to bind to the angiotensin-converting-enzyme 2 (ACE2) receptor on target cells. In some cases, the resulting anti-idiotype antibodies can mimic the Spike protein itself, thus binding ACE2 and exerting either agonist or antagonist effects or inducing a complement-mediated attack on ACE-2-expressing cells (19).

Limited data suggest a potential overlap in symptoms between post-acute COVID-19 syndrome (PACS) and post-COVID-19 vaccination syndrome (PCVS) (20).

While limited data exists, direct comparisons between patients exhibiting clinically overlapping syndromes stemming from SARS-CoV-2 infection and COVID-19 vaccination are lacking. We herein present a paired sequential analysis of patients presenting with similar neurological manifestations with central nervous system (CNS) or peripheral nervous system (PNS) involvement associated with SARS-CoV-2 infection or COVID-19 vaccination.

Moreover, we sought to investigate the presence of anti-idiotype antibodies in these patients, as their presence could potentially indicate an ongoing autoimmune process contributing to the neurological manifestations observed. The temporal associations presented in our case series suggest that SARS-CoV-2 infection and COVID-19 vaccination may have triggered similar immune-mediated disorders of the peripheral and central nervous systems in the presence of anti-idiotype antibodies directed against the ACE2 protein.

## 2 Patients and methods

### 2.1 Patients

Samples were collected and evaluated during routine follow-up after providing informed consent. All samples were cryopreserved at -20°C until use. Clinical data was collected from either inpatient or outpatient clinical reports.

### 2.2 Immunofluorescence assay

Anti-nuclear antigens (ANA), anti-neutrophilic cytoplasmic antibodies (ANCA), aquaporin 4 (AQP4), myelin oligodendrocyte glycoprotein (MOG), and onconeural antibodies were investigated using immunofluorescence assay (IFI; Hep-2, mosaic Hep-2 and granulocytes, HEK transfected cells substrate and primate cerebellum, respectively; Euroimmun, Lübeck, Germany, in accordance with the manufacturer’s instructions).

### 2.3 Onconeural antibodies immunoblot assay

Onconeural antibodies (anti-Hu, Yo, Ri, amphiphysin, CRMP5, Ma2, and GAD) were detected by immunoblot using recombinant onconeural proteins (Ravo Diagnostika, Freiburg, Germany).

### 2.4 Antibodies anti-ENA, anti dsDNA assay

ENA (RNP, Sm, Ro60, La, Scl-70, Jo-1, and centromere protein B) were investigated in the patients’ serum by fluorescence enzyme immunoassay (FEIA, Thermo Fisher, Uppsala, Sweden), while anti-dsDNA was investigated using chemiluminescent immunoassay (CLIA, Werfen Barcelona, Spain) in accordance with the manufacturer’s instruction.

### 2.5 Antibodies anti-ACE2 ELISA assay

The serum concentration of anti-ACE2 antibodies was evaluated using a commercial ELISA assay (EAGLE Biosciences, USA). Briefly, the serum from patients was incubated with a coated microtiter plate, followed by horseradish peroxidase (HRP)-labeled anti-human IgG secondary reagent. The microplate was then read using Analyzer I-2P (Euroimmun, Lübeck, Germany), where the color intensity of the substrate reaction was correlated with the serum concentration of anti-ACE2 antibodies.

## 3 Case descriptions

### 3.1 PNS clinical syndrome

#### 3.1.1 Post-SARS-CoV-2 vaccine

In November 2021, a 38-year-old woman, presented to the emergency department (ED) 7 days after receiving the first dose of the Moderna anti-SARS-CoV-2 vaccine, complaining of right lower limb and back pain.

Neurological examination revealed weak deep tendon reflexes, distal burning paresthesia, and dysesthesia in the lower limbs. As laboratory testing disclosed an elevated D-Dimer (340.000 mg/L; reference value <500 mg/L), body CT scan and brain MRI were performed, with unremarkable results. The spine MRI demonstrated contrast enhancement (CE) in the proximal portion of nerve roots D12 to L5 bilaterally; no CE was detected in the spinal cord or in the cauda roots. Nerve conduction study (NCS) disclosed absent F-waves in the deep peroneal nerve bilaterally, while motor/sensory conduction velocity and nerve potential amplitude resulted normal in the four limbs. Rachycentesis disclosed 11 cells/mmc, normal protein and glucose CSF levels, no oligoclonal bands, and negative polymerase chain reaction (PCR) assay for neurotropic viruses (EBV, CMV, HSV1, HSV2, and VZV). On serum TPHA, VDRL, *Borrelia burgdorferi*, HIV, toxoplasma screening, rheumatoid factor, anti-citrullinated protein antibodies, ANA, ANCA, ENA, anti-dsDNA, and anti-ganglioside antibodies all tested negative. Both the clinical presentation and the CSF findings did not meet the criteria for Guillain–Barré Syndrome (21). Anti-ACE2 antibodies tested positive on serum (35.76 U/mL; cutoff, 30 U/mL). Within a span of 2 months, after immunomodulating therapy with intravenous methylprednisolone (500 mg/day for 5 days) followed by oral tapering over a month, the patient experienced an improvement of back pain and of dysesthesia up to their disappearance. Spine MRI at 2 months’ follow-up demonstrated resolution of the previously reported CE, posing the suspicion of a monophasic post-COVID-19-vaccine immune-mediated radiculitis with anti-ACE2 antibodies.

#### 3.1.2 Post-SARS-CoV-2 infection

In October 2021, a 60-year-old male was admitted to the ER for dyspnea due to COVID-19 interstitial pneumonia and treated with high-flow oxygen therapy. Then, 20 days after testing negative for SARS-CoV-2 on transcription real-time PCR (rRT-PCR) assay, he developed tingling and pricking paresthesia in distal upper and lower limbs, muscle cramps, and gait imbalance. Neurological examination showed mild distal weakness in the four limbs, tactile hypoesthesia and paresthesia with stocking–glove distribution, gait ataxia, and global areflexia. The CSF analysis disclosed albuminocytological dissociation, and NCS showed features consistent with demyelinating sensory-motor polyneuropathy. Thus, a diagnosis of GBS was made (21). Anti-ganglioside antibodies all tested negative. Anti-ACE2 antibodies were positive on serum (35.22 U/mL; cutoff, 30 U/mL). The patient experienced clinical improvement after a course of plasmapheresis.

### 3.2 CNS clinical syndromes

#### 3.2.1 Post-SARS-CoV-2 vaccine

In February 2021, an 80-year-old man with a history of ischemic heart disease and atrial fibrillation developed lower limb tactile hypoesthesia and rigidity 3 days after receiving the first dose of anti-COVID-19 vaccine (Pfizer). Then, 1 month later, he received the second dose of the vaccine, reporting progressive worsening of gait due to a sense of stiffness in the lower limbs. Neurological examination disclosed exaggerated deep tendon reflexes in the lower limbs, clonus of the right ankle, pyramidal hypertonia in the right lower limb, and spastic–ataxic gait, without sensory levels or Lhermitte’s sign.

The motor evoked potentials, nerve conduction studies, and brain MRI result were normal; spine MRI disclosed multiple cervical and lumbar disc herniation without radicular compression or spinal cord abnormalities. Blood chemistry test ruled out vitamin B12 or folate deficiency. Due to the patient’s concurrent anticoagulant therapy for atrial fibrillation, a spinal tap was not performed. Autoantibodies against AQP-4, MOG, GlyR, GAD, and amphiphysin, as well as onconeural antibodies, all tested negative. Antibodies anti-ACE2 tested positive on serum (37.33 U/mL; cutoff, 30 U/mL).

Post-COVID-19 vaccine MRI-negative myelitis associated with ACE2 autoantibodies was then suspected. Despite therapy with intravenous methylprednisolone (500 mg/day for 5 days) followed by oral tapering, there was no evidence of clinical improvement.

At a 24-month follow-up, the paraparetic ataxic gait remains unaltered, thereby corroborating the hypothesis of a previous acute monophasic illness.

#### 3.2.2 Post-SARS-CoV-2 infection

In August 2022, a 57-year-old-male presented with fever and pharyngodynia due to SARS-CoV-2 infection. Then, 15 days after testing negative for SARS-CoV-2 on rRT-PCR assay, he started to experience bilateral lower limb pain, episodic spasms, and gait instability that led to frequent falls. The patient also reported urinary incontinence. Given the progressive worsening of symptoms up to the inability of walking independently, the patient was admitted to the Neurology Department. Neurological examination revealed proximal weakness and diffuse bilateral pyramidal hypertonia in the lower limbs, prominent on the right side, hyperreflexia in the lower limbs, persistent right ankle clonus, and paraparetic–spastic gait requiring bilateral support for walking. The cervical, thoracic, and lumbar spine MRIs did not show any pathological contrast enhancement or abnormal cord signal; the brain MRI was normal. Autoantibodies against AQP-4, MOG, GlyR, GAD, and amphiphysin, as well as onconeural antibodies, all tested negative. Computed tomography (CT) imaging of the chest and abdomen ruled out malignancy.

A CSF analysis disclosed only mild elevation in protein level (810 mg/L; reference value 200–400 mg/L). Both electromyography and nerve conduction velocities resulted normal; cortical somatosensory evoked potentials (SSEPs) and motor evoked potentials (MEPs) disclosed impaired central conduction in the lower limbs bilaterally, with prominent MEP alteration in the right upper and lower limb. Anti-ACE2 antibodies tested positive on serum (37.47 U/mL; cutoff, 30 U/mL).

Given the clinical, laboratory, and instrumental findings, post-infectious immune-mediated MRI-negative myelitis was suspected. Therapy with intravenous methylprednisolone (500 mg/day for 5 days) followed by oral tapering and a course of intravenous immunoglobulin (IVIg) (2 g/kg over 5 days) was promptly administered. The patient did show some clinical improvement after receiving immunomodulatory therapy, but at 12-month follow-up the spastic paraparetic gait persists, requiring single (rather than double) support.

## 4 Discussion and conclusion

Emerging evidence, including case reports and small case series, suggests a potential association between SARS-CoV-2-infection and also SARS-CoV-2 vaccination and small fiber neuropathy, a debilitating peripheral neuropathy (8–11, 22).

Our patients exhibited overlapping neurological manifestations following SARS-CoV-2 infection or vaccination, involving either the peripheral nervous system (PNS) or central nervous system (CNS) (radiculitis, Guillain-Barré syndrome, and MRI-negative myelitis, respectively) while sharing positivity for anti-ACE2 antibodies (the main features are summarized in **Table 1**).

**Table 1:** Comparison of clinical, instrumental, and laboratory characteristics of patients.

Patient no.	Age and sex	Trigger	Time from the onset of symptoms	CNS or PNS clinical syndrome (diagnosis)	Clinical features	Spine MRI	EDX study	CSF analysis		Anti-ACE2 antibodies (U/mL)^c^	Therapy
								Ly (/µL)^a^	Prot (g/L)^b^		
1	38, F	Anti-SARS-CoV-2 vaccine	7 days after vaccine injection	PNS (immune mediated radiculitis)	Weak DTR in LL, distal burning paresthesia and dysesthesia in LL	CE in the proximal portion of nerve roots D12 to L5 bilaterally	NCS: absent F-waves in deep peroneal nerve bilaterally	11	0.40	37.76	Methylprednisolone
2	60, M	SARS-CoV-2 infection	20 days after negative rRT-PCR assay	PNS (GBS)	Distal weakness in UL and LL, hypoesthesia and paresthesia with stocking-glove distribution, gait ataxia, and global areflexia	NP	NCS: demyelinating sensory-motor polyneuropathy in UL and LL	4	0.85	35.22	PEX
3	80, M	Anti-SARS-CoV-2 vaccine	30 days after vaccine injection	CNS (MRI-negative myelitis)	Neurological examination disclosed exaggerated deep tendon reflexes in the lower limbs, clonus of the right ankle, pyramidal hypertonia in the right lower limb, and spastic-ataxic gait, without sensory levels or Lhermitte’s sign	Spine MRI: no spinal cord abnormalities	Normal MEPs and NCS	NP	NP	37.33	Methylprednisolone
4	57, M	SARS-CoV-2 infection	15 days after negative rRT-PCR assay	CNS (MRI-negative myelitis)	Proximal weakness and bilateral pyramidal hypertonia in LL, hyperreflexia in LL, R ankle clonus, and paraparetic–spastic gait	Brain MRI: unremarkable; spine MRI: no spinal cord abnormalities	EMG and NCS: normal; SSEPs and MEPs: impaired central conduction in LL limbs bilaterally (prominent MEPs alteration in the R UL and LL)	3	0.81	37.47	IVIg§

ACE2, angiotensin-converting-enzyme 2; CE, contrast enhancement; CNS, central nervous system; CSF, cerebrospinal fluid; DTR, deep tendon reflexes; EDX, electrodiagnostic; EMG, electromyography; F, female; GBS, Guillain–Barré syndrome; IVIg, intravenous immunoglobulins (§2 g/kg over 5 days); LL, lower limbs; Ly, lymphomonocytes; M, male; MEPs, motor evoked potentials; MRI, magnetic resonance imaging; NCS, nerve conduction studies; NP, not performed; PEX, plasma exchange; PNS, peripheral nervous system; Prot, total proteins; R, right; rRT-PCR, transcription real-time PCR; SSEPs, cortical somatosensory evoked potentials; UL, upper limbs.

a Reference range, <5.

b Reference range, <0.52.

c Cutoff, 30 U/mL.

MRI-negative myelitis is reported to be associated with anti-MOG autoantibodies (23), acute flaccid myelitis, idiopathic transverse myelitis, and paraneoplastic disorders of the spine (24, 25). Only two cases of spastic paraparesis ascribed to MRI-negative myelitis associated with COVID-19 infection were reported so far (26, 27). In contrast, both COVID-19-related and post-COVID-19 vaccine GBS have been reported (6, 12, 13).

PACS and PCVS seem to share the same etiopathogenesis; however, the current definition of post-COVID-19 vaccine neurologic pathology is, in part, elusive, lacking objective evaluations to support identification (28). There are multiple pathogenetic mechanisms that have been hypothesized to be responsible for the neurological symptoms of PACS (29); these include SARS-CoV-2 neurotropism and neuroinvasion through ACE2 receptor (30), endothelial disruption (31), viral-induced coagulopathy (32), neurovascular injury (33), and persistent systemic inflammation with cytokine storm (34). Although a role of the immune system dysregulation has been proposed (35), the underlying mechanisms have not yet been clarified and include expansion of monocyte subsets and T cell dysregulation (36, 37), leading to blood–brain barrier impairment and neuroglial dysfunction (38). It has also been hypothesized that the production of autoantibodies plays a role in the abnormal immune response that endures after infection (39); in this context, the antibody production is thought to be generated by the strong immune and inflammatory response rather than the virus itself (40). The detected autoantibodies comprise antibodies against cytokines (41), ACE2 (42), and ANA (43). The chronic dyspnea, exhaustion, and brain fog seen in PACS have been related to persistent ANA autoreactivity (44).

The association between COVID-19 vaccine and autoimmune phenomena remains subject of debate (45), and the understanding of its underlying molecular mechanisms remains nebulous. Nonetheless, specific autoantibody generation (46), molecular mimicry (47), and the involvement of certain vaccination adjuvants (48, 49) have all been proposed as causal factors in the pathophysiology of PCVS (4, 50).

Our data reveal clinically overlapping syndromes triggered by SARS-CoV-2 infection or vaccination. While we hypothesized an immune-mediated mechanism underlying these neurological manifestations, we were unable to detect a “classical” autoimmune profile in these patients. The autoantibodies against AQP-4, MOG, GlyR, GAD, amphiphysin, and onconeural antibodies for CNS syndromes and anti-ganglioside antibodies for PNS syndromes were all negative in these patients. On the contrary, an analysis of the serum of these four patients revealed the sole immune-mediated reaction to be directed against the ACE2 protein.

Supporting the proposed immune-mediated mechanism involving SARS-CoV-2 stimulation and anti-idiotype generation, anti-ACE2 antibodies have been previously identified in patients with connective tissue diseases like systemic lupus erythematosus (SLE) and scleroderma (51). These antibodies appear to inhibit ACE2 activity, potentially contributing to the development of constrictive vasculopathies (52).

Murphy and Longo (19) speculated on the existence of anti-idiotype antibodies against ACE2 that may mediate adverse events following SARS-CoV-2 infection or vaccination against it, resulting in the activation of the immune system against cells expressing ACE2. According to this hypothesis, Ab2 antibodies can also cause neurologic adverse effects, given the expression of ACE2 on neuronal tissue and the specific process of SARS-CoV-2 invasion of the CNS (53, 54). Arthur and colleagues (42) demonstrated the actual presence of anti-ACE2 antibodies in 81% of patients who have recovered from COVID-19 infection, thus having formed antibodies against the RBD of SARS-CoV-2: this corroborates the hypothesis that these are, in fact, anti-idiotype antibodies. Anti-ACE2 antibodies are not found in the serum of patients that have not been infected. Furthermore, these antibodies decrease the plasmatic activity of soluble ACE2, leading to increased angiotensin II and subsequent activation of the immune system and inflammatory response.

In the present case series, anti-ACE2 could not be determined to be causative, from a pathogenetic point of view. However, its presence in the absence of other classic autoimmune markers of CNS or PNS involvement suggests that anti-idiotypes and additional anti-idiotype markers may be helpful to support the care of patients with spike-associated neuropathies.

Our observations suggest a clinically overlapping pattern of neurological manifestations involving both the central nervous system (CNS) and peripheral nervous system (PNS) following SARS-CoV-2 infection and vaccination in susceptible patients. It is plausible that such manifestations have a non-classical autoimmune etiology, potentially linked or associated to the development of anti-idiotype antibodies directed against the angiotensin-converting enzyme 2 (ACE2) protein. This autoimmune response may be triggered by both vaccination and natural infection, particularly in individuals with heightened or misdirected antigen-specific immune responses.

In a multiomics study, Su and colleagues correlated the presence of specific anti-ACE2 anti-idiotype antibodies together with other factors with the early identification of COVID-19 patients who would develop PASC (55). Other authors report on the development of anti-ACE2 autoantibodies after SARS-CoV-2 infection. Indeed many patients with a history of SARS-CoV-2 infection have antibodies specific for ACE2. Patients with anti-ACE2 antibodies have a lower activity of soluble plasmatic ACE2, and plasma from these patients seems to inhibit exogenous ACE2 activity. These findings are consistent with the hypothesis that anti-ACE2 antibodies develop after SARS-CoV-2 infection and reduce the plasmatic ACE2 activity. This could lead to an increase in the abundance of angiotensin II, which causes a proinflammatory state that triggers the symptoms of PASC (42, 56).

When considering this previous evidence, the present findings of anti-idiotype (anti-ACE2) in both PASC-vax and PASC (Long-COVID) patients with overlapping neurological presentation and symptoms indicate that a common pathway to the PASC inflammatory syndrome is present. In this context, it remains to be determined whether anti-ACE2 antibodies could mechanically underlie the genesis of the neurological damage or rather parallel the inflammatory condition leading to PASC. In this context, this possibility is confirmed by evidence of persistence of viral RNA after SARS-CoV-2 vaccination that could generate prolonged protein production and immune stimulation in some selected patient reservoirs (57–59).

Additionally, studies have demonstrated the persistence of SARS-CoV-2 spike protein fragments in peripheral blood monocytes (60). These studies have all shown that SARS-CoV-2 spike protein fragments can persist in peripheral blood monocytes for months or even years after infection. This persistence could contribute to the development of long-term symptoms of COVID-19, such as fatigue, neurological problems, and cardiopulmonary dysfunction. These observations support the hypothesis that prolonged stimulation of the immune system might lead to a distorted and persistent immune response, potentially contributing to the development of autoimmunity, possibly also associated with anti-ACE2 antibodies. However, the pathogenesis in only a fraction of infected or vaccinated patients and the pathogenetic role of anti-ACE2 antibodies remain to be conclusively demonstrated in large-scale, structured studies.

Lastly, it is imperative to acknowledge some limitations pertaining to our study. Literature on the subject is still lacking at present, and our data refers to a small number of patients. Additionally, the pandemic status that led to the infection of countless individuals and called for the implementation of a large-scale vaccination program emphasizes the need to consider the ethical issues that apply to the evaluation of such patients.

## Patients’ perspective

Our four patients and their families were very concerned about the occurrence of neurological symptoms associated with COVID-19 infection and particularly frustrated when the symptoms were associated with vaccination. However, they were relieved to learn that there were therapies available to treat their condition, trusting that early therapy could be beneficial. Although recovery was not complete in all patients, eventually they were all grateful to the neurologists who had cured them and glad to agree to share their case, understanding that their condition and the associated implications could be useful for further research.

## Data availability statement

The original contributions presented in the study are included in the article/supplementary material. Further inquiries can be directed to the corresponding author.

## Ethics statement

Written informed consent was obtained from the individual(s) for the publication of any potentially identifiable images or data included in this article.

## Author contributions

MB: Conceptualization, Data curation, Investigation, Writing – original draft. FB: Methodology, Writing – review & editing. CC: Writing – review & editing. GP: Methodology, Supervision, Writing – review & editing. AB: Writing – review & editing. AH: Investigation, Writing – review & editing. AL: Writing – review & editing. AU: Writing – review & editing. LB: Conceptualization, Data curation, Funding acquisition, Investigation, Methodology, Supervision, Writing – original draft, Writing – review & editing. AM: Conceptualization, Methodology, Supervision, Validation, Writing – review & editing.
